# Daily Variation in the Occurrence of Different Subtypes of Stroke

**DOI:** 10.1155/2017/9091250

**Published:** 2017-06-22

**Authors:** Luciana Ripamonti, Roberto Riva, Fabiola Maioli, Corrado Zenesini, Gaetano Procaccianti

**Affiliations:** ^1^IRCCS Institute of Neurological Sciences, Bologna, Italy; ^2^Department of Biomedical and Neuromotor Sciences, University of Bologna, Bologna, Italy; ^3^Department of Medicine, Maggiore Hospital, Bologna, Italy

## Abstract

Three thousand two hundred and ninety-eight patients admitted to our Stroke Unit with hemorrhagic, large artery atherosclerosis, cardioembolic, small-vessel occlusion, and undetermined etiology-cryptogenic strokes were included in the study. The circadian variability in onset in each stroke subgroup and the associations with various risk factors were analyzed. In each subgroup, a significant minority of patients suffered from stroke during sleep. In the ischemic group, hypercholesterolemia, paroxysmal atrial fibrillation, and previous myocardial infarction facilitated the onset during waking. During waking, stroke onset was significantly higher in the morning compared to the afternoon both in the hemorrhagic and in the ischemic type. In hemorrhagic stroke, a previous stroke was associated with a lower early morning occurrence. In large artery atherosclerosis stroke, males were at higher risk of early morning occurrence (*p* < 0.01). In small-vessel occlusion stroke, hypertension is significantly more present in the morning compared to the afternoon onset (*p* < 0.005). Circadian patterns of stroke onset were observed both in hemorrhagic and in ischemic stroke, irrespective of the ischemic subgroup. In all groups, stroke was more likely to occur during waking than during sleep and, in the diurnal period, during morning than during afternoon. Moreover, sex and some clinical factors influence the diurnal pattern.

## 1. Introduction

Circadian variation in stroke onset has been analyzed since the early 1970s, but until the beginning of the new millennium data were very discordant [1, 2]. In 1998 a meta-analysis [3] revealed a higher frequency of onset stroke in the morning with a lower risk during the night. In the last 20 years other studies have contributed more information especially in highlighting some differences in the various etiological subtypes. However, some data remain discordant [4–31] (see Table 1 for details). Moreover, risk factors have been given little consideration in relation to daily onset in stroke subtypes and mostly without significant results [4, 5, 10, 17, 32–37]. However, most authors have not studied the subtype simultaneously or, when done, the number of patients was too small to investigate the difference in circadian onset or the association between circadian rhythms and risk factors in the various stroke subtypes.

The aim of our study was to investigate the circadian characteristics in stroke subtypes. Moreover, we analyzed the association among rhythms and some risk factors.

## 2. Patients and Methods

Our results are based on a retrospective, single-center study of consecutive patients admitted to the Stroke Unit of the Institute for Neurological Disease of Bologna, Italy, over 10 years (January 2004–December 2013). All patients admitted to the Stroke Unit were recruited for the study. The study was approved by the local ethical committee (approval 272/CE, project 16021). The Stroke Unit is situated within the Institute of Neurological Sciences, with a catchment area of about 270,000–320,000 residents. A consecutive series of 4,560 patients were recruited. The registration form consists of demographic and vital variables, stroke subtype, date and hour of onset, situation at onset, symptoms and clinical findings, family history, history of hypertension, diabetes, hypercholesterolemia, neurological, psychiatric, and cardiovascular disease, use of drugs, functional grade, and clinical evolution. Stroke criteria were defined according to the World Health Organization. Ischemic strokes were classified in line with the TOAST classification. Diagnosis of stroke was made in the presence of rapidly emerging focal or global neurological signs with fast progression, lasting at least 24 h or leading to death without apparent cause other than a vascular origin. Patients admitted were classified into the following subtypes: large artery atherosclerotic stroke (AT), cardioembolic stroke (CE), lacunar stroke (LA), cryptogenic stroke (CRY), and intracerebral hemorrhagic stroke, other cerebrovascular events or etiology, and transient ischemic attack. Patients with subarachnoid hemorrhage were not admitted to our clinic unit and so they were not recruited for the study.

Stroke onset time was defined as the time at which the patient or a witness first noted clear neurological signs. Determination of the stroke onset time was possible in 3,689 patients. The impairment caused by the stroke was objectively evaluated by the National Institutes of Health Stroke Scale (NIHSS). The degree of disability was measured according to the modified Rankin Score (mRS) [47, 48].

### 2.1. Statistical Analysis

Continuous variables are presented as mean and standard deviation (SD) while categorical variables as absolute frequency (percentage). ANOVA and chi-square were used to compare variables among subtypes of stroke. The survival curves were estimated with the Kaplan-Meier method and were compared with log-rank test of equality of survivor functions. Hazard ratios (HR) and 95% confidence intervals (95% CI) are presented. Differences in the proportions of stroke during the diurnal period were performed with time of onset divided into three-hour intervals (8–11, 11–14, 14–17, and 17–20) and assessed by the proportion test. Multivariate logistic or univariate linear regressions were performed to study the association between clinical variables and different diurnal period and between awake and asleep onset. On multivariate analyses models, age, gender, previous stroke, hypertension, diabetes mellitus, previous myocardial infarction, hypercholesterolemia, permanent or acute atrial fibrillation, prestroke modified Rankin Score, and admission NIHHS were modelled as independent variables while awake/asleep or diurnal time interval were modelled as dependent variables. Odds ratio (OR), slope (*β*), and 95% CI are reported when appropriate. Statistical analysis was performed using Statistical Package Stata SE, version 14.0 [49].

## 3. Results

### 3.1. Population

Data from 4,560 subjects were recorded. Five hundred patients with other cerebrovascular events or etiology and 762 with stroke but with unknown onset time were excluded from the analysis. Our study groups comprised the remaining 3,298 patients with stroke. The principal variables in each subtype are shown in Table 2.

Age and sex did not differ between hemorrhagic and all ischemic strokes, while they significantly differed among ischemic subtypes. As expected, the other clinical variables significantly differed between ischemic and hemorrhagic stroke and among ischemic subgroups.

### 3.2. Asleep/Awake Onset

At first, we analyzed the distribution of stroke occurrence during sleep and during awake periods. In the sleep period we included patients that became aware of the stroke on awakening in which we did not know the exact hour of onset.

About one-quarter (770 out of 3,298 patients) of stroke patients suffered from stroke during sleep (*p* < 0.001 sleep onset versus asleep onset). This pattern was observed in each stroke subgroup (Table 2).

Except for a slightly less seniority in the whole ischemic group with asleep onset compared to awake onset (OR = 0.98, 95% CI = 0.97–0.99), no other demographic data were different between awake and asleep onset, while some pathophysiological characteristics modified the awake/asleep onset pattern in some subgroups (Table 3). In the whole ischemic group, hypercholesterolemia (OR = 1.28, 95% CI = 1.03–1.60), paroxysmal atrial fibrillation (OR = 1.70, 95% CI = 1.14–2.55), and previous myocardial infarction (OR = 1.38, 95% CI = 1.03–1.86) facilitated the awake onset. The main differences observed in hypercholesterolemia are substantially related to the CRY subgroup (OR = 1.67, 95% CI 1.10–2.49). On the contrary, in the whole ischemic group, the fact of having had a previous stroke is associated with asleep onset (OR = 0.77, 95% CI = 0.60–0.99). This difference is due to CE (OR = 0.62, 95% CI = 0.40–0.98) and LA (OR = 0.62, 95% CI = 0.18–0.98). Paroxysmal atrial fibrillation is associated with asleep onset in the hemorrhagic group (OR = 0.35, 95% CI = 0.14–0.82). No other clinical factor is associated with awake/asleep onset period in other subgroups.

No association of the admission NIHSS and prestroke mRS was observed with awake or asleep onset. The awake/asleep onset period is however associated with different degrees of mRS at hospital discharge in some subgroups. Ischemic stroke showed a worse disability mRS (*β* = 0.14, 95% CI = 0.02–0.26, and *p* = 0.032) in asleep onset. This difference is associated with fibrinolysis (*β* = 0.41 ± 0.10, 95% = CI = 0.21–0.62, *p* < 0.001), which was performed in ischemic patients if the hospital arrival time was soon after stroke onset. In these subgroups fibrinolysis was associated with a better discharge mRS (*β* = 0.39, 95% CI = 0.01–0.778, and *p* = 0.047 in CE and 0.55, 95% CI = 0.86–0.23, and *p* = 0.001 in CRY). Hemorrhagic stroke showed a slightly worse disability score at discharge in awake onset (*β* = 0.30, 95% CI = 0.10–0.55, and *p* = 0.043) compared to asleep onset. No clinical variables were correlated with these features.

**Figure 1:**
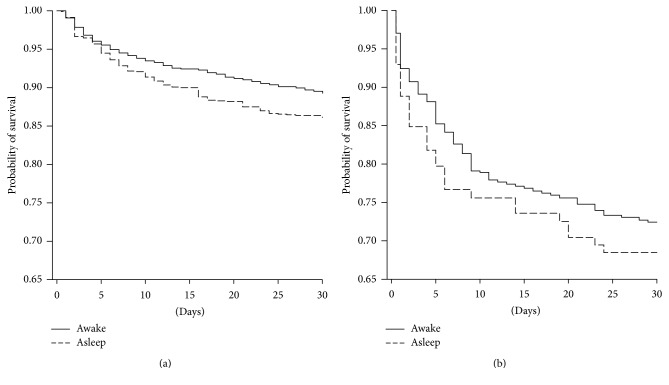
Short-term survival probability (Kaplan-Meier estimates) between awake and asleep onset in (a) ischemic and (b) hemorrhagic stroke. (a) = *p* = 0.025; (b) = ns.

**Figure 2:**
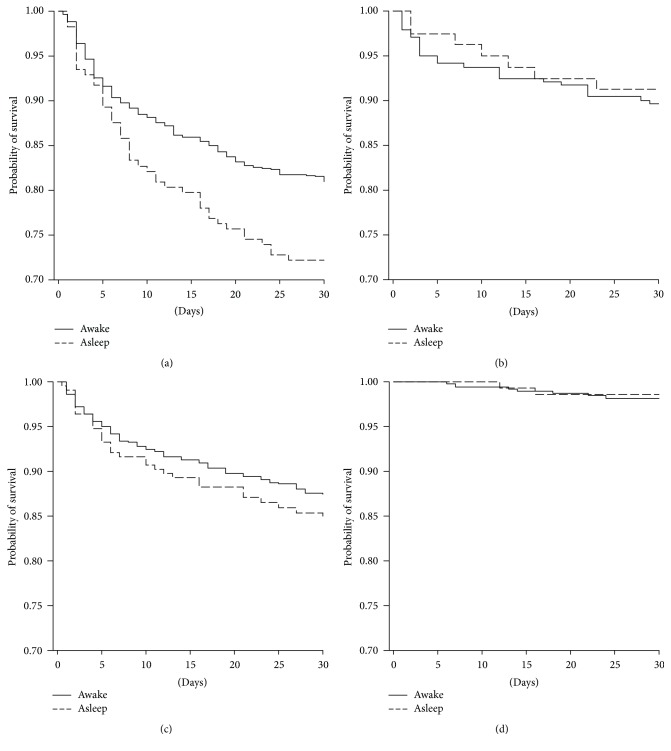
Short-term survival probability (Kaplan-Meier estimates) between awake and asleep onset in (a) CE, (b) AT, (c) CRY, and (d) LA stroke. (a) = *p* = 0.01 and (b), (c), and (d) = ns.

Asleep onset ischemic stroke patients had a 1.33 higher risk of death within the first month of stroke compared to awake onset patients (HR = 1.33, 95% CI = 1.04–1.72, Figure 1(a)). This difference is due to the CE subgroups, in which this risk is 1.55 higher in asleep compared to awake onset (HR = 1.55, 95% CI = 1.10–2.19) (Figure 2(a)). This difference was not associated with fibrinolysis.

No difference in death rate within the first month was observed in hemorrhagic stroke (HR = 1.18, 95% CI = 0.79–1.76, Figure 1(b)) or in the other groups (Figures 2(b), 2(c) and 2(d)).

### 3.3. Diurnal Onset

**Figure 3:**
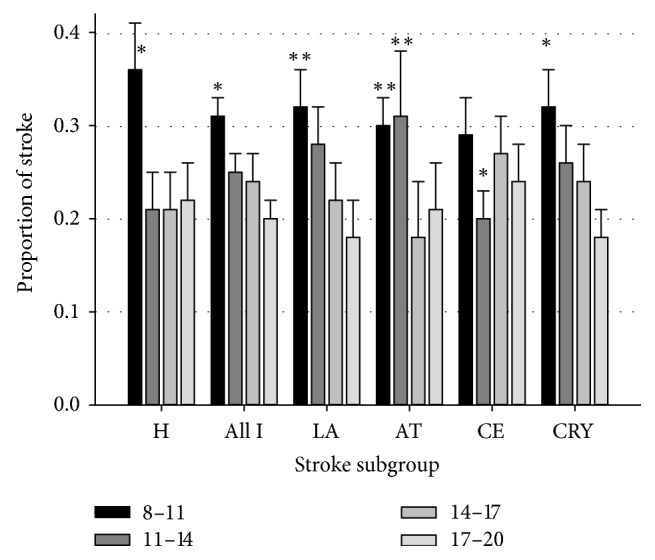
Proportion of stroke onset during diurnal period in subgroups of stroke. H, hemorrhagic; all I, all ischemic; LA; AT; CE; CRY. Black bars = 8–11; dark gray bars = 11–14; gray bars = 14–17; light gray bars = 17–20. *∗* indicates at least *p* < 0.005 from other intervals; *∗∗* indicates at least *p* < 0.01 from afternoon intervals.

During the diurnal period stroke onset is significantly more frequent in the morning compared to the afternoon, irrespective of stroke subtype (Figure 3). However, some difference in the patterns can be observed among subtypes: hemorrhagic, LA, and CRY had a more frequent onset in the first part of the morning (*p* < 0.001), while AT had a more frequent onset throughout the whole morning (*p* < 0.01) and CE exhibited a significantly lower frequency of onset in the late morning (*p* < 0.001). Demographic variables and risk factors were associated in a different way with circadian onset in different stroke subtypes (Table 4).

Patients with hemorrhagic stroke in case of at least one previous stroke experienced less frequently the stroke onset in the early morning than during other diurnal periods (14% versus 39%, OR = 0.15, and 95% CI = 0.04–0.50), while hypercholesterolemia was 2.36 times less frequent in the late afternoon (17:00–20:00) onset (11% versus 26%, OR = 0.33, and 95% CI = 0.14–0.79). Sex influenced the time of onset in AT patients (OR = 2.66, 95% CI = 1.30–5.44): male patients showed a higher risk and female patients a lower risk for early morning onset compared to afternoon onset.

In LA, arterial blood pressure influenced onset times (OR = 2.25, 95% CI = 1.30–3.88): hypertensive patients showed a higher onset frequency in the early morning compared to other diurnal times while normotensive patients had almost half of the risk for early morning onset compared to other diurnal periods.

None of the other prognostic variables studied were associated with onset time in other subgroups of stroke.

CRY patients with early morning onset exhibited a slightly better admission NIHSS score compared with other times of onset (OR = 0.96, 95% CI = 0.94–0.99, Table 4).

## 4. Discussion 

Our study, including more than 3,000 patients, confirms that both ischemic and hemorrhagic stroke occur preferentially during waking and in certain specific periods of diurnal time in accordance with most authors [1–46] (Table 1). This may depend on pathophysiological factors partially shared by patients with ischemic and hemorrhagic stroke.

Different physiological and pathological factors and different lifestyles may be responsible for and influence the onset of stroke during sleep or waking. In particular, patients with ischemic stroke that occurred during sleep were more likely to have had at least one previous stroke and to be older than patients with ischemic stroke that occurred during waking. On the contrary, in patients with a concomitant cardiovascular disease, as shown by hypercholesterolemia, previous myocardial infarction, and atrial fibrillation, the onset of ischemic stroke is likely to occur during waking.

Moreover, our study suggests, for the first time, that the time of stroke occurrence may correlate with prognosis and outcome, with differences between ischemic and hemorrhagic stroke. Patients with ischemic stroke that occurred during sleep display a worse disability level at hospital discharge compared to ischemic stroke occurred during waking and have a higher risk of death during the first month. However, no difference in the prestroke disability presence was observed. Discharge disability score was strongly due to fibrinolysis therapy performed in about 10% of awake onset CE and CRY patients. However, 30-day mortality was not influenced by fibrinolysis therapy. Our data on worse discharge disability and great mortality risk in ischemic stroke occurring during sleep are in agreement with published data [22] but we added the new information that, in ischemic stroke, this is true only for the CE group.

Hemorrhagic stroke patients with awake onset showed a worse disability level at hospital discharge but the 30-day mortality was not significantly different compared to asleep onset. Our data did not agree with published data in which authors suggested worse conditions in hemorrhagic stroke with asleep compared to awake onset [11]. However, the latter is performed on a small number of patients. A recent multicenter study reports that patients arriving in the emergency department at night or in the morning with “idiopathic” intraparenchymal hemorrhage had higher in-hospital mortality than those arriving in the afternoon [38]. Our data show a slight even if not significant increase of mortality at one month from stroke, but, as can be observed in Figure 1(b), an increase in mortality during the first and possibly in-hospital period can be suggested in our patients too.

Awake/asleep onset seems to influence prognosis in different ways in hemorrhagic and ischemic stroke since awake onset influences negatively discharge disability in hemorrhagic and positively in ischemic stroke. We observed that in our ischemic patients this is due to fibrinolysis therapy. Moreover, in the same ischemic patients we found a lower mortality during the first month after stroke in awake onset irrespective of fibrinolysis therapy.

Therefore, a new interesting feature was that awake/asleep onset seems to be in some way associated with outcome. To our knowledge this is the first report showing a correlation between outcome and awake/asleep time of occurrence in stroke subgroups.

As concerns the diurnal pattern of onset, our findings are consistent with previous studies [2, 3] in observing that stroke onset had a diurnal pattern even when controlling for other considered variables. In all strokes there is a significantly higher risk of occurrence in the morning and a lower risk in the late afternoon. This is also true in hemorrhagic stroke, in which this pattern is controversially discussed in the literature. In particular all subtypes of studied stroke occur preferably in the first few hours after awakening in accordance with many authors (Table 1). Some authors have reported two peaks of onset, one in the morning and the other in the afternoon for some subtype of strokes [2, 4–6, 8, 19, 20, 25, 29, 32, 34, 35] but our data did not confirm this hypothesis. In the same way we did not confirm data on higher night onset in some subgroups as reported by other, few, authors [23, 39–42]. Moreover, we observed a specific diurnal time pattern in each stroke subgroup. Previous data considering onset time in specific subgroups of patient were discordant and were performed on a small group of patients; our study contributes useful information.

It is interesting to note that, in hemorrhagic stroke patients, the fact of having had at least one previous stroke seems to be protective for the early morning onset compared to other diurnal periods, while hypercholesterolemia seems to favor stroke during the morning and early afternoon compared to the late afternoon. However, since these data are not corrected for oral anticoagulant and anticholesterol therapies, no conclusions can be drawn. Sex influenced diurnal onset. Males suffered significantly more frequently from stroke in the early morning than in the afternoon, while on the contrary females were less affected in the early morning than in the afternoon.

In LA patients, in which hypertension is an important predisposing factor, being normotensive protects patients from developing stroke during the highly at-risk awake period.

The pattern we observed in these cerebrovascular accidents has already been observed in other acute cardiovascular events [50–56]. This pattern parallels the reported diurnal variation in blood pressure [57], which is higher in the morning, and higher blood pressure is accepted as a risk factor for stroke. Other different risk factors such as coagulability, epinephrine and norepinephrine, and alpha-sympathetic vasoconstriction are higher in the morning [6, 58]. Moreover, platelet aggregation increases significantly in the morning during the assumption of an upright posture and when beginning the daily activities [59, 60]. Daily variability in stroke occurrence can reflect autonomic nervous system modulations and even more its modifications with aging, which is suspected to facilitate stroke. Vagal tonic modulation during the night, in fact, is higher in young males and decreases with aging and it has been associated with stroke risk [61, 62]. These differences may partially justify the different circadian distribution of stroke onset between males and females and during aging. Our data suggest that the daily occurrence of stroke is significantly influenced by the circadian clock that determines biorhythms, the circadian fluctuations of vital and physiological parameters, and the subjects' activities.

Also other recurrent critical events in other diseases, not necessarily vascular ones, such as chronic airway disease [63], rheumatoid arthritis [64], allergic rhinitis [64], epileptic seizures [60], headache attacks [65], and psychiatric disorders [66] among others, may be affected by internal biorhythms. The roles played by the circadian clock in pathologies deserve to be studied more extensively and possibly to be given better consideration for prophylaxis and treatment purposes.

## 5. Limitations 

The present study has different limitations. It is a retrospective study based on data collected during hospitalization that included only patients with AT, CE, CRY, LA, or intracerebral hemorrhage stroke and thus information on other etiologies is lacking. The main limitation of this study was, however, not having considered some factors such as therapy taken before and during hospitalization, presence of disruption of circadian rhythms, subjects' activities, or other circumstances temporally close to the stroke onset as the possible effect of nocturnal blood pressure and the extreme seasonal and house humidity and temperature.

## 6. Conclusions

Our study has the strength of considering the stroke onset in relation to patient characteristics in a large cohort of patients drawn from a well-defined catchment area. An advantage is that we considered all consecutive patients admitted to a Stroke Unit over a period of 10 years. In all cases considered there was indication of precise stroke onset and if it was reported on awakening we considered it as asleep onset. Thus, we were able to consider stroke onset in relation to waking/sleep alternation as well.

Our study confirms that stroke as many other cardiovascular diseases occurs preferentially during waking and in the morning irrespective of the subtype. We observed that some risk factors are associated with time of stroke onset, suggesting that they interact with biorhythms in inducing stroke. In this context, strategies considering the specific treatment of risk factors during the circadian period of their major influence on stroke onset may ameliorate preventive and therapeutic interventions. Further efforts to study the specific aspects of circadian rhythms on cerebrovascular disease are needed to better understand the pathophysiological features and to obtain beneficial effects in terms of prevention and treatment.

## Figures and Tables

**Table 1:** Available evidence on circadian pattern of onset of stroke.

Ref.	Author, year	Country	Cases (*n*)	Peak	Notes
[2]	Manfredini et al., 2005	Italy	—	Morning and early evening	Review

[3]	Elliott, 1998	USA	IS, IH, and SAH 11816	06:00–12:00	Meta-analysis

[4]	Stergiou et al., 2002	Greece	IS + IH = 811	06:00–12:00 and 16:00–20:00	>50 and <81 yrs

[5]	Casetta et al., 2002	Italy	IS = 1395	08:00–09:00 and 20:00–21:00	

[6]	Omama et al., 2006	Japan	IS = 7575, IH = 3852	IS 06:00–12:00, IH 06:00–12:00, and 16:00–20:00	

[7]	Inagawa et al., 2000	Japan	IH = 267	Awake	

[8]	Butt et al., 2009	Pakistan	IS = 438, IH = 329	IS 04:00–08:00 and 16:00–20:00, IH 08:00–12:00	

[9]	Chaturvedi et al., 1999	USA	AT = 173,	AT and CE 06:00–12:00, LA 12:00–18:00	The onset more frequent during sleep was LA
			CE = 210,		
			LA = 210		

[10]	Passero et al., 2000,	Italy	IH = 901	06:00–12:00	Onset peak due to hypertensive IH

[11]	Nagakane et al., 2006	Japan	IH = 129	Awake	

[12]	Anderson et al., 2004	New Zealand	IS and IH = 1497	06:00–12:00	

[13]	Uddin et al., 2015	Bangladesh	IS = 50	06:00–12:00	

[14]	Naess et al., 2011	Norway	AT = 80, CE = 191, LA = 136, IH = 662	LA 00:00–06:00, IH 06:00–18:00	

[15]	Lago et al., 1998	Spain	LA = 209, CE = 228, AT = 429	09:00–10:00	

[16]	Spengos et al., 2005	Greece	IS = 1216, IH = 232		First-ever stroke
					Onset more frequent during sleep was LA

[17]	Bornstein et al., 1999	Israel	IS = 1671	Awake	

[18]	Cheung et al., 2001	Hong Kong	IS = 608, IH = 177	IS 06:00–12:00, IH 06:00–18:00	

[19]	Spengos et al., 2003	Greece	AT = 171, CE = 406, LA = 227, IH = 200	06:00–12:00, 16:00–18:00	

[20]	Spengos et al., 2003	Greece	CE = 300	08:00–10:00	
				16:00–18:00	

[21]	Turin et al., 2013	Japan	IS = 897, IH = 335	Awake	

[22]	Jiménez-Conde et al., 2007	Spain	IS = 813	09:00–12:00	

[23]	Kocer et al., 2005	Turkey	IS = 917, IH = 240	IS 03:00–06:00	
				IH, ns	

[24]	Nyquist et al., 2001	USA	IH = 85	08:00–16:00	

[25]	Feng et al., 2011	USA	IH = 215	10:00–12:00 and 18:00–20:00	

[26]	Choi et al., 2015	Korea	AT = 256, LA = 276, CE = 155	06:00–12:00	

[27]	Fodor et al., 2014	Romania	IS = 969, IH = 94	06:00–12:00	

[28]	Fodor et al., 2014	Romania	AT = 60, CE = 153, LA = 538	06:00–12:00	

[29]	Inagawa, 2003	Japan	IH = 350	Men <70 yrs 08:00–10:00 and 18:00–20:00	
				All women or men >69 yrs 18:00–20:00	

[30]	Serena et al., 2003	Spain	IS = 1248	06:00–12:00	

[31]	Bassetti and Aldrich, 1999	Switzerland	IS = 65	08:00–12:00	

[32]	Turin et al., 2009	Japan	IH = 637	08:00–10:00 and 20:00–21:00	

[33]	Tsementzis et al., 1985	UK	IS = 245 IH = 118	10:00–12:00	All <70 yrs

[34]	Wroe et al., 1992	UK	IS = 545, IH 66	06:00–12:00, IS second peak at 14:00–16:00	

[35]	Sloan et al., 1992	USA	IH = 237	10:00–12:00, a second peak at 18:00–20:00	

[36]	Argentino et al., 1990	Italy	IS = 426	06:00–10:00	

[38]	Fabbian et al., 2016	Italy	CH = 517	Female 08:00–10:00, male 12:00–14:00	Both idiopathic and posttraumatic cerebral hemorrhage were included

[39]	Marshall, 1977	UK	Nonembolic IS = 554, IH = 153	IS 00:00–06:00, female IH 06:00–12:00	Sex difference in time course
					Embolic IS were not included

[40]	Caplan et al., 1983	USA	IS = 127	Asleep in thrombotic and awake in embolic stroke	

[41]	Arboix and Martí-Vilalta, 1990	Spain	IS = 142 (69 AT, 45 LA, 28 CE); IH = 33	AT 00:00–00:06, IH 00:06–12:00	Difference in onset among different etiologies
				CE 06:00–18:00, LA ns	

[42]	Hossmann, 1971	Germany	IS = 131	01:00–05:00	

[43]	Pasqualetti et al., 1990	Italy	IS = 508, IH = 159	IS morning, IH ns	

[44]	Marler et al., 1989	USA	IS = 1167	08:00–10:00	

[45]	Marsh et al. 1990	USA	IS = 151	06:00–10:00	

[46]	Ricci et al., 1992	Italy	IS = 375, IH = 375	06:00–12:00	The IS more frequent during sleep was LA

IS = ischemic stroke. IH = intracerebral hemorrhagic stroke. AT = large artery atherosclerotic stroke; CE = cardioembolic stroke; CRY = cryptogenic stroke; LA = lacunar stroke.

**Table 2:** Principal characteristics of patients in relation to stroke subtype. Differences between groups were evaluated by chi-square with Yate correction for continuity or with *t*-test when appropriate.

	Hemorrhagic stroke (*n* = 543)	Ischemic stroke (*n* = 2755)	*p* value between the two main groups	AT (*n* = 345)	CE (*n* = 762)	CRY (*n* = 858)	LA (*n* = 790)	*p* value among ischemic subtypes
Age mean (SD)	75.3 (11.9)	75.7 (11.5)	0.461	74.0 (10.4)	79.9 (9.8)	73.8 (12.8)	74.4 (11.0)	<0.001
Sex, M *n*(%)	279 (51.4)	1418 (51.5)	0.460	231 (67.0)	311 (40.8)	414 (48.3)	462 (58.5)	<0.001
Awake onset *n*(%)	439 (80.8)	2089 (75.8)	0.092	263 (76.2)	577 (75.7)	657 (76.6)	592 (74.9)	0.888
Previous stroke *n*(%)	72 (13.3)	411 (14.9)	0.351	53 (15.4)	126 (16.5)	118 (13.8)	114 (14.4)	0.442
Hypertension *n*(%)	394 (72.6)	1826 (66.3)	0.005	219 (63.5)	439 (57.6)	559 (65.2)	609 (77.1)	<0.001
Diabetes mellitus *n*(%)	101 (18.6)	681 (24.7)	0.003	97 (28.1)	160 (21.0)	196 (22.8)	228 (28.9)	<0.001
Previous myocardial infarction *n*(%)	49 (9.0)	339 (12.3)	0.036	53 (15.4)	104 (13.6)	100 (11.7)	82 (10.4)	0.063
Hypercholesterolemia *n*(%)	113 (20.8)	672 (24.4)	0.083	97 (28.1)	150 (19.7)	227 (26.5)	198 (25.1)	0.003
Permanent atrial fibrillation *n*(%)	54 (9.9)	470 (17.1)	<0.001	9 (2.6)	389 (51.0)	42 (4.9)	30 (3.8)	<0.001
Paroxysmal atrial fibrillation *n*(%)	25 (4.6)	323 (11.7)	<0.001	6 (1.7)	260 (34.1)	38 (4.4)	19 (2.4)	<0.001
mRS admission ≥ 1 *n*(%)	195 (35.9)	967 (35.1)	0.754	97 (28.1)	331 (43.4)	300 (35.0)	239 (30.3)	<0.001
NIHSS scale admission mean (SD)	14.8 (12.7)	8.8 (8.8)	<0.001	10.3 (8.4)	12.7 (10.2)	8.9 (8.8)	4.2 (3.9)	<0.001
Number of patients with fibrinolysis	0	214	—	28 (8.1)	54 (7.1)	104 (12.1)	28 (3.5)	<0.001

mRS = modified Ranking Score; NIHHS = National Institute of Health Stroke Scale. AT = large artery atherosclerotic stroke; CE = cardioembolic stroke; CRY = cryptogenic stroke; LA = lacunar stroke.

**Table 3:** Multivariate logistic regression between clinical variables (independent variables) and awake/asleep stroke onset (dependent variable).

	Hemorrhagic	All ischemic	CE	AT	CRY	LA
	OR 95%CI					
Age	0.99	0.98	0.98	0.97	0.99	0.99
	0.97–1.01	0.97–0.99	0.96–1.00	0.95–1.00	0.97–1.00	0.98–1.01
Sex	0.86	1.06	0.81	1.10	1.25	1.18
	0.54–1.36	0.88–1.29	0.57–1.16	0.64–1.91	0.89–1.76	0.83–1.68
Previous stroke	0.57	0.77	0.62	0.95	1.05	0.62
	0.32–1.02	0.60–0.99	0.40–0.98	0.45–2.00	0.65–1.73	0.18–0.98
Hypertension	1.11	0.97	1.07	0.98	0.83	1.05
	0.69–1.80	0.79–1.17	0.76–1.52	0.56–1.71	0.58–1.19	0.71–1.55
Diabetes mellitus	0.82	1.03	1.07	0.86	1.11	1.02
	0.48–1.41	0.84–1.27	0.69–1.62	0.38–1.52	0.75–1.64	0.70–1.48
Previous myocardial infarction	1.29	1.38	1.68	1.80	1.25	1.07
	0.58–2.86	1.03–1.86	0.96–2.94	0.78–4.17	0.72–2.17	0.60–1.90
Hypercholesterolemia	0.93	1.28	0.77	1.44	1.67	1.37
	0.55–1.59	1.03–1.60	0.50–1.20	0.77–2.70	1.10–2.49	0.92–2.10
Permanent atrial fibrillation	1.23	0.96	0.94	1.17	0.94	1.20
	0.60–2.56	0.72–1.29	0.64–1.38	0.23–6.06	0.46–1.92	0.47–3.09
Paroxysmal atrial fibrillation	0.35	1.70	1.60	0.93	1.32	3.45
	0.14–0.82	1.14–2.55	0.98–2.63	0.10–8.77	0.48–3.61	0.78–15.15
Prestroke mRS	0.67	0.94	0.85	1.14	0.75	1.25
	0.41–1.09	0.76–1.15	0.58–1.26	0.62–2.12	0.51–1.09	0.83–1.87
Admission NIHSS	0.98	0.99	0.99	1.02	1.00	1.01
	0.97–1.00	0.98–1.00	0.97–1.00	0.99–1.05	0.98–1.02	0.97–1.06

mRS = modified Ranking Score; NIHHS = National Institute of Health Stroke Scale; AT = large artery atherosclerotic stroke; CE = cardioembolic stroke; CRY = cryptogenic stroke; LA = lacunar stroke.

**Table 4:** Multivariate logistic regression among demographic variables and risk factors (both independent variables) with daytime onset in different stroke subtypes. 8–11 versus all other periods (dependent variable).

	Hemorrhagic	CE	AT	CRY	LA
	OR 95% CI				
Age	0.99	1.00	1.02	1.01	0.99
	0.97–1.02	0.96–1.03	0.98–1.06	0.99–1.02	0.98–1.02
Sex (M versus F)	0.81	0.93	2.66	1.09	0.92
	0.48–1.35	0.59–1.47	1.30–5.44	0.72–1.65	0.58–1.46
Previous stroke	0.15	1.13	0.45	1.28	1.38
	0.04–0.50	0.60–2.13	0.18–1.16	0.70–2.32	0.76–2.52
Hypertension	0.80	0.88	1.92	0.82	2.25
	0.47–1.35	0.57–1.37	0.97–3.80	0.53–1.26	1.30–3.88
Diabetes mellitus	1.78	1.11	1.10	1.03	0.93
	0.96–3.30	0.66–1.87	0.52–2.32	0.64–1.65	0.59–1.47
Previous myocardial infarction	0.69	0.56	0.84	0.96	0.93
	0.26–1.82	0.29–1.11	0.31–2.29	0.52–1.79	0.59–1.47
Hypercholesterolemia	1.39	1.07	0.75	1.35	0.90
	0.76–2.55	0.62–1.84	0.36–1.57	0.85–2.14	0.55–1.46
Permanent atrial fibrillation	1.42	1.37	0.11	0.69	2.48
	0.60–3.37	0.84–2.20	0.01–1.32	0.24–2.03	0.91–6.77
Paroxysmal atrial fibrillation	0.54	1.15	NE	0.18	0.93
	0.10–2.85	0.66–2.01		0.02–1.42	0.31–2.75
Prestroke mRS	1.12	1.28	2.15	1.28	0.86
	0.64–1.95	0.77–2.13	0.94–4.92	0.79–2.08	0.53–1.41
Admission NIHSS	1.01	1.01	0.98	0.96	1.03
	0.99–4.99	0.99–1.04	0.01–3.05	0.94–0.99	0.97–1.08

NE = not estimable for lack of cases in at least one condition. mRS = modified Ranking Score; NIHHS = National Institute of Health Stroke Scale. AT = large artery atherosclerotic stroke; CE = cardioembolic stroke; CRY = cryptogenic stroke; LA = lacunar stroke.
